# Supplementary figures and images for: New deep data hiding and extraction algorithm using multi-channel with multi-level to improve data security and payload capacity
Source: PeerJ Comput Sci. 2022 Oct 19;8:e1115. doi: 10.7717/peerj-cs.1115 (PMC9680874; doi:10.7717/peerj-cs.1115)

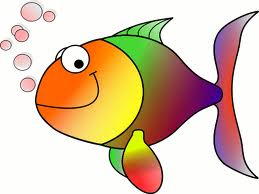

Supplement: Supplemental Information 2 — Copyright 2019 Mohammad Shamim Imtiaz. [file peerj-cs-08-1115-s002.zip › standard-test-images-for-Image-Processing-a096eed0c535bf7c772d6662799e37683c207179/standard_test_images/HappyFish.jpg]

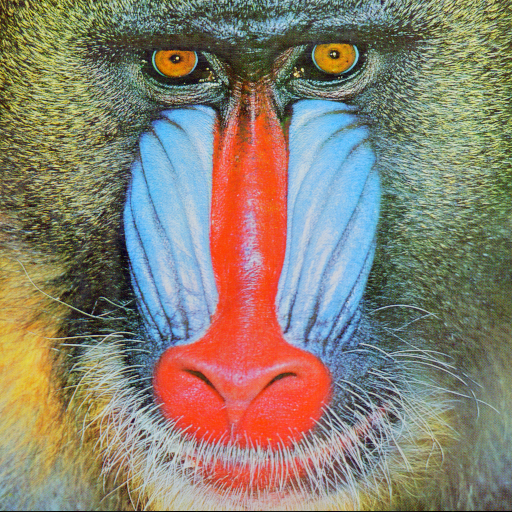

Supplement: Supplemental Information 2 — Copyright 2019 Mohammad Shamim Imtiaz. [file peerj-cs-08-1115-s002.zip › standard-test-images-for-Image-Processing-a096eed0c535bf7c772d6662799e37683c207179/standard_test_images/baboon.png]

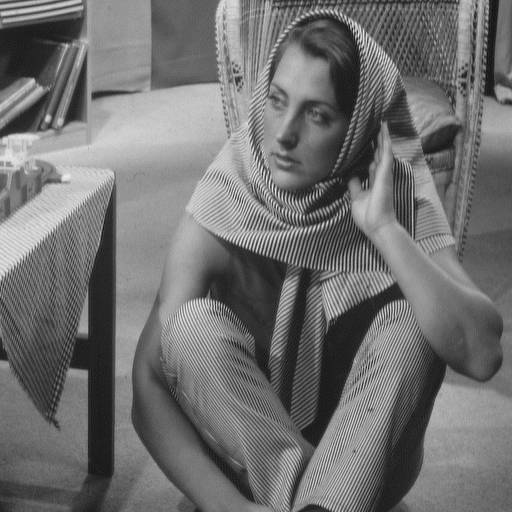

Supplement: Supplemental Information 2 — Copyright 2019 Mohammad Shamim Imtiaz. [file peerj-cs-08-1115-s002.zip › standard-test-images-for-Image-Processing-a096eed0c535bf7c772d6662799e37683c207179/standard_test_images/barbara.bmp]

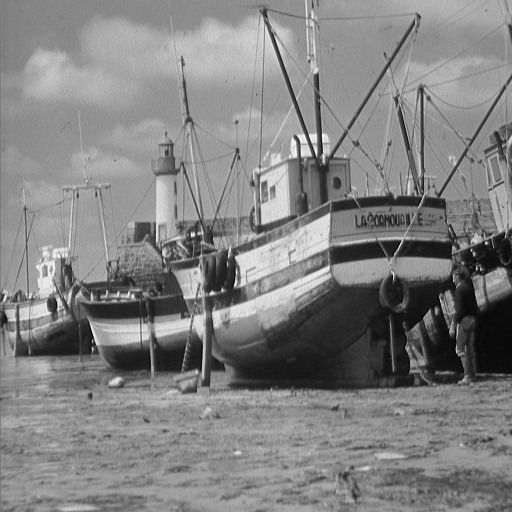

Supplement: Supplemental Information 2 — Copyright 2019 Mohammad Shamim Imtiaz. [file peerj-cs-08-1115-s002.zip › standard-test-images-for-Image-Processing-a096eed0c535bf7c772d6662799e37683c207179/standard_test_images/boat.png]

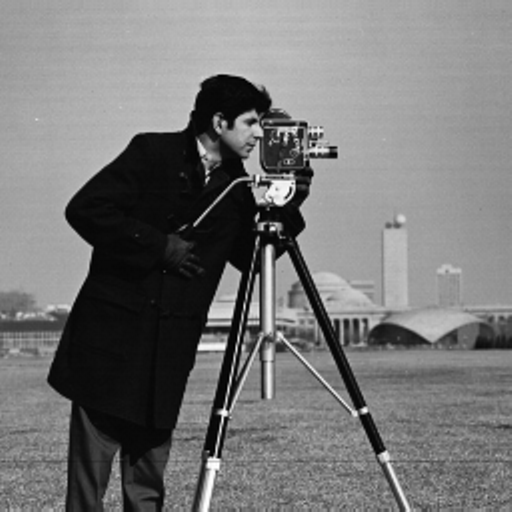

Supplement: Supplemental Information 2 — Copyright 2019 Mohammad Shamim Imtiaz. [file peerj-cs-08-1115-s002.zip › standard-test-images-for-Image-Processing-a096eed0c535bf7c772d6662799e37683c207179/standard_test_images/cameraman.tif]

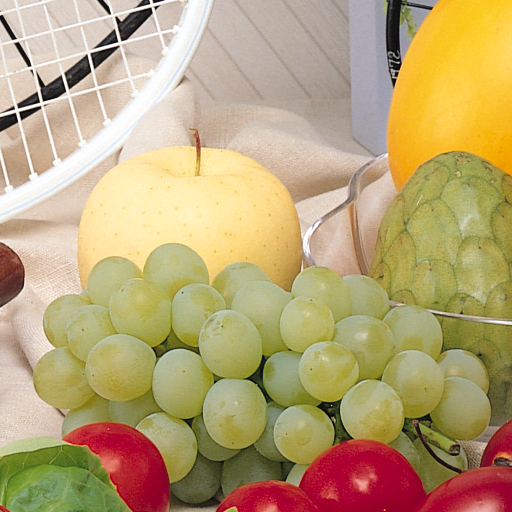

Supplement: Supplemental Information 2 — Copyright 2019 Mohammad Shamim Imtiaz. [file peerj-cs-08-1115-s002.zip › standard-test-images-for-Image-Processing-a096eed0c535bf7c772d6662799e37683c207179/standard_test_images/fruits.png]

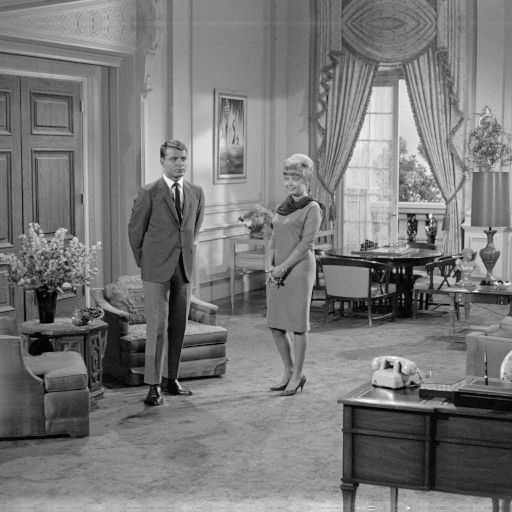

Supplement: Supplemental Information 2 — Copyright 2019 Mohammad Shamim Imtiaz. [file peerj-cs-08-1115-s002.zip › standard-test-images-for-Image-Processing-a096eed0c535bf7c772d6662799e37683c207179/standard_test_images/livingroom.tif]

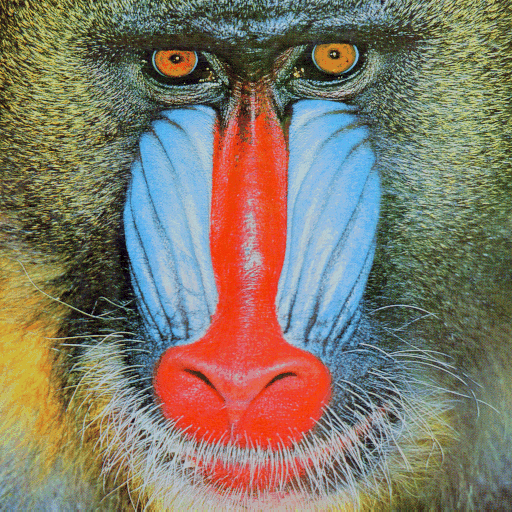

Supplement: Supplemental Information 2 — Copyright 2019 Mohammad Shamim Imtiaz. [file peerj-cs-08-1115-s002.zip › standard-test-images-for-Image-Processing-a096eed0c535bf7c772d6662799e37683c207179/standard_test_images/mandril_color.tif]

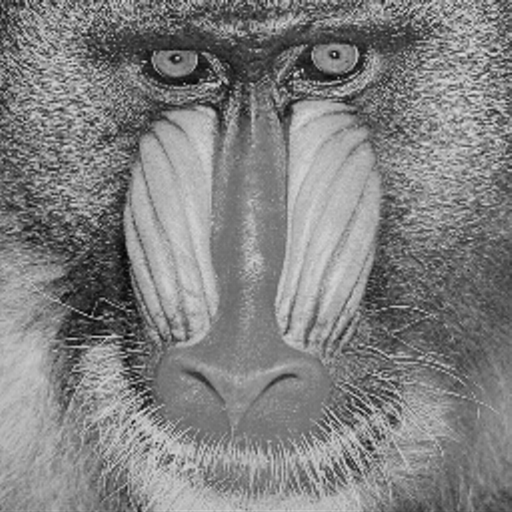

Supplement: Supplemental Information 2 — Copyright 2019 Mohammad Shamim Imtiaz. [file peerj-cs-08-1115-s002.zip › standard-test-images-for-Image-Processing-a096eed0c535bf7c772d6662799e37683c207179/standard_test_images/mandril_gray.tif]

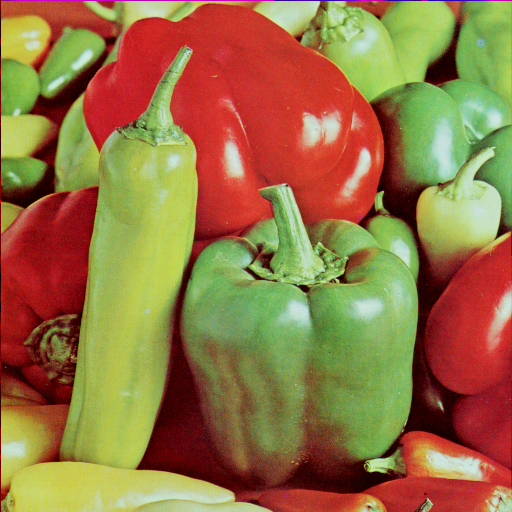

Supplement: Supplemental Information 2 — Copyright 2019 Mohammad Shamim Imtiaz. [file peerj-cs-08-1115-s002.zip › standard-test-images-for-Image-Processing-a096eed0c535bf7c772d6662799e37683c207179/standard_test_images/peppers.png]

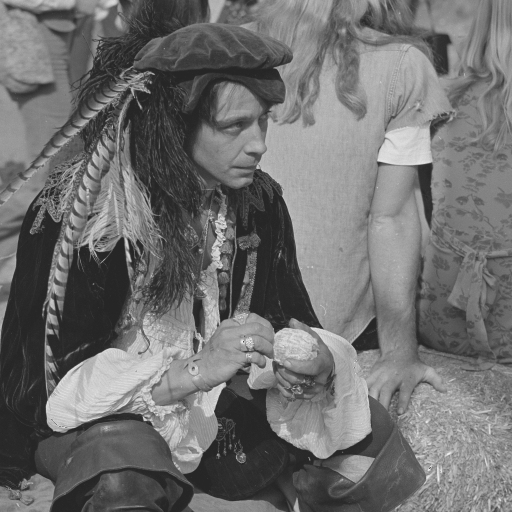

Supplement: Supplemental Information 2 — Copyright 2019 Mohammad Shamim Imtiaz. [file peerj-cs-08-1115-s002.zip › standard-test-images-for-Image-Processing-a096eed0c535bf7c772d6662799e37683c207179/standard_test_images/pirate.tif]

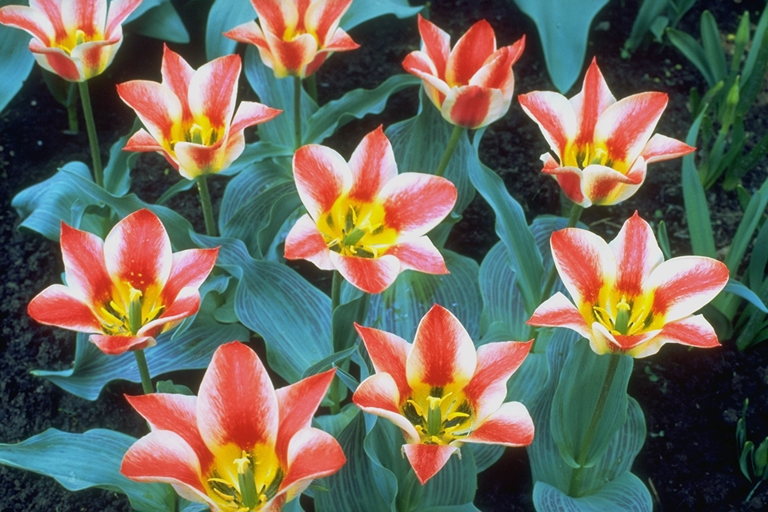

Supplement: Supplemental Information 2 — Copyright 2019 Mohammad Shamim Imtiaz. [file peerj-cs-08-1115-s002.zip › standard-test-images-for-Image-Processing-a096eed0c535bf7c772d6662799e37683c207179/standard_test_images/tulips.png]

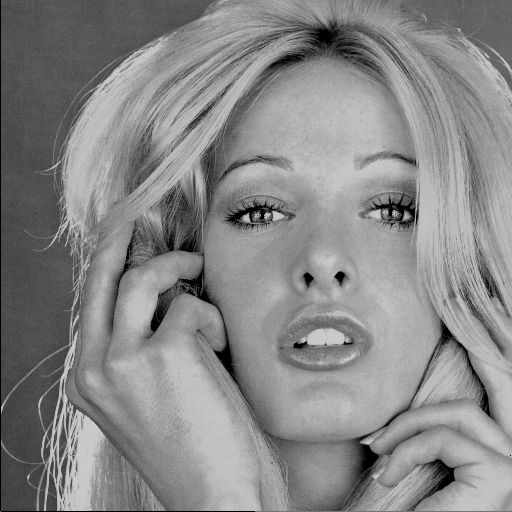

Supplement: Supplemental Information 2 — Copyright 2019 Mohammad Shamim Imtiaz. [file peerj-cs-08-1115-s002.zip › standard-test-images-for-Image-Processing-a096eed0c535bf7c772d6662799e37683c207179/standard_test_images/woman_blonde.tif]

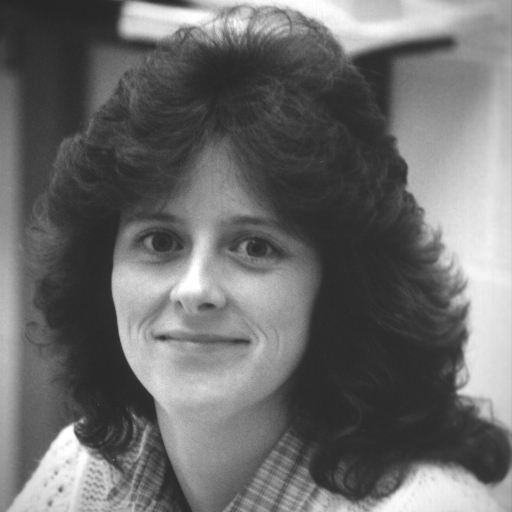

Supplement: Supplemental Information 2 — Copyright 2019 Mohammad Shamim Imtiaz. [file peerj-cs-08-1115-s002.zip › standard-test-images-for-Image-Processing-a096eed0c535bf7c772d6662799e37683c207179/standard_test_images/woman_darkhair.tif]

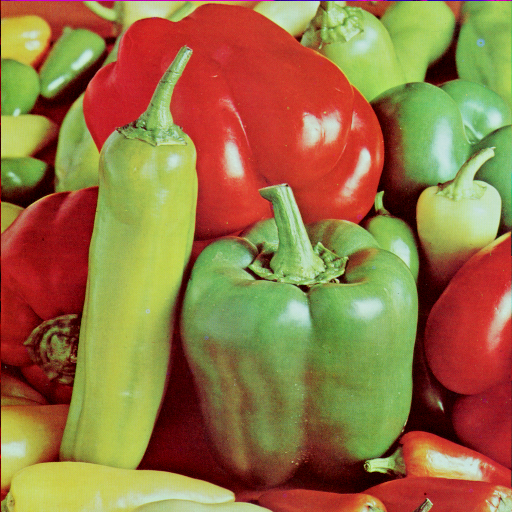

Supplement: Supplemental Information 5 — Copyright 2019 Mohammad Shamim Imtiaz. [file peerj-cs-08-1115-s005.zip › Stego_image_Lena_Baboon_Pepper/PVD_stego_Pepper 105000.bmp]

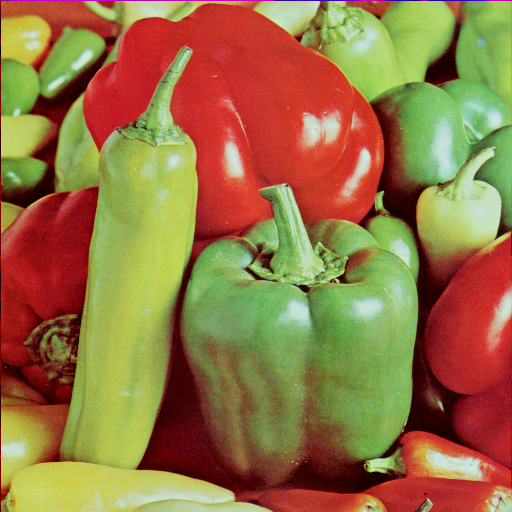

Supplement: Supplemental Information 5 — Copyright 2019 Mohammad Shamim Imtiaz. [file peerj-cs-08-1115-s005.zip › Stego_image_Lena_Baboon_Pepper/PVD_stego_Pepper 40000.bmp]

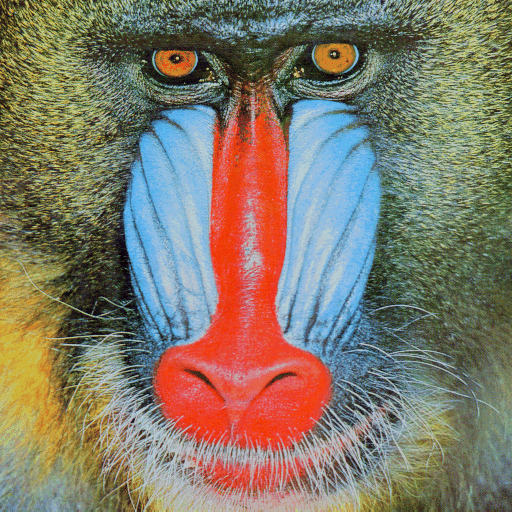

Supplement: Supplemental Information 5 — Copyright 2019 Mohammad Shamim Imtiaz. [file peerj-cs-08-1115-s005.zip › Stego_image_Lena_Baboon_Pepper/PVD_stego_image_Baboon_30.bmp]

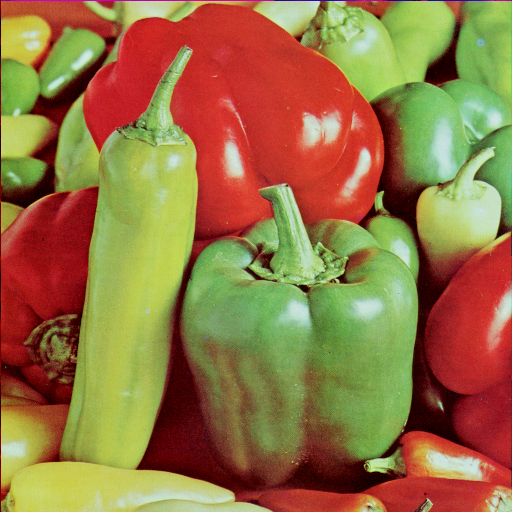

Supplement: Supplemental Information 5 — Copyright 2019 Mohammad Shamim Imtiaz. [file peerj-cs-08-1115-s005.zip › Stego_image_Lena_Baboon_Pepper/PVD_stego_Pepper 80000.bmp]

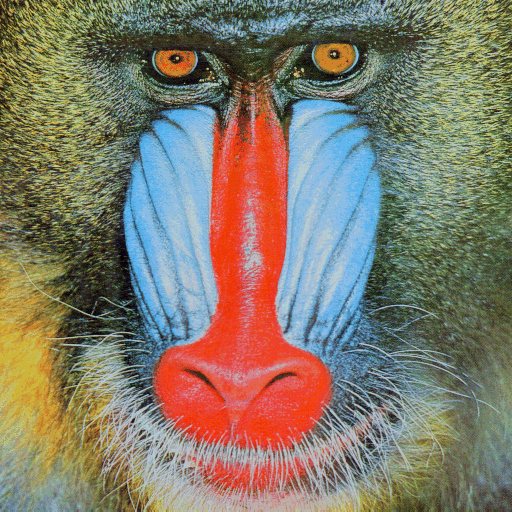

Supplement: Supplemental Information 5 — Copyright 2019 Mohammad Shamim Imtiaz. [file peerj-cs-08-1115-s005.zip › Stego_image_Lena_Baboon_Pepper/PVD_stego_image_Baboon_50.bmp]

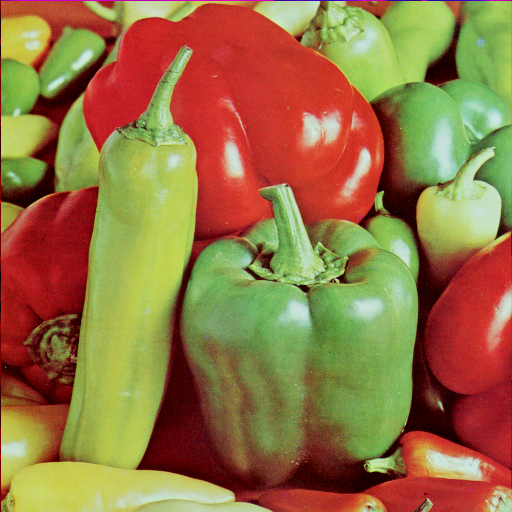

Supplement: Supplemental Information 5 — Copyright 2019 Mohammad Shamim Imtiaz. [file peerj-cs-08-1115-s005.zip › Stego_image_Lena_Baboon_Pepper/PVD_stego_image_Pepper_10.bmp]

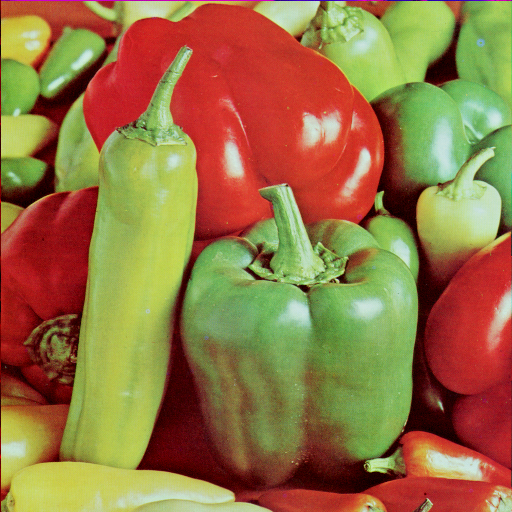

Supplement: Supplemental Information 5 — Copyright 2019 Mohammad Shamim Imtiaz. [file peerj-cs-08-1115-s005.zip › Stego_image_Lena_Baboon_Pepper/PVD_stego_image_Pepper_40.bmp]

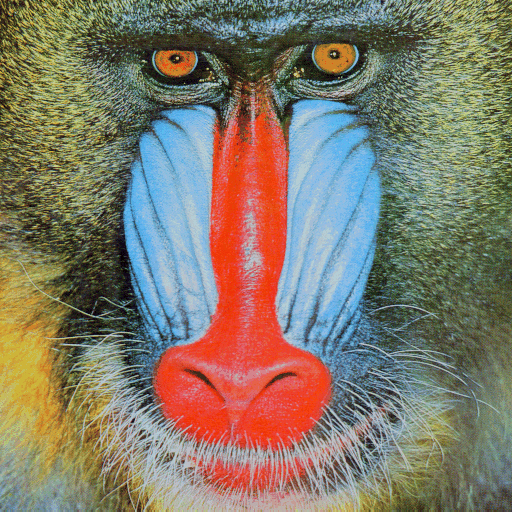

Supplement: Supplemental Information 5 — Copyright 2019 Mohammad Shamim Imtiaz. [file peerj-cs-08-1115-s005.zip › Stego_image_Lena_Baboon_Pepper/PVD_stego_image_Baboon_40.bmp]

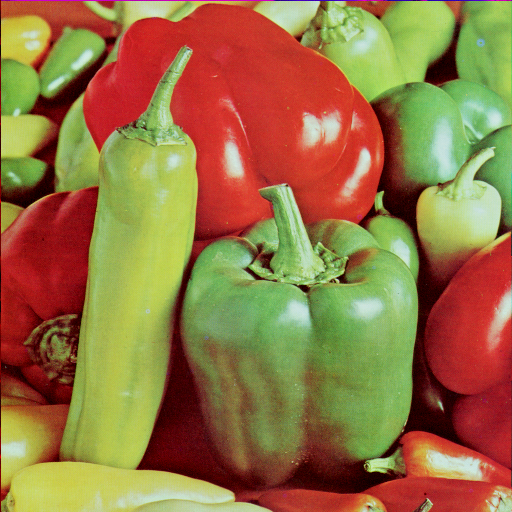

Supplement: Supplemental Information 5 — Copyright 2019 Mohammad Shamim Imtiaz. [file peerj-cs-08-1115-s005.zip › Stego_image_Lena_Baboon_Pepper/PVD_stego_Pepper 20000.bmp]

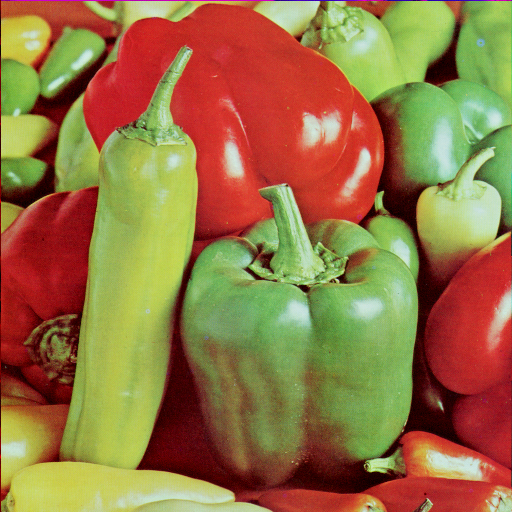

Supplement: Supplemental Information 5 — Copyright 2019 Mohammad Shamim Imtiaz. [file peerj-cs-08-1115-s005.zip › Stego_image_Lena_Baboon_Pepper/PVD_stego_image_Pepper_50.bmp]

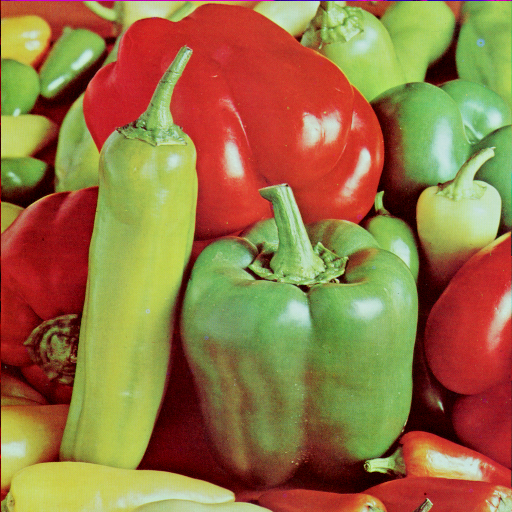

Supplement: Supplemental Information 5 — Copyright 2019 Mohammad Shamim Imtiaz. [file peerj-cs-08-1115-s005.zip › Stego_image_Lena_Baboon_Pepper/PVD_stego_image_Pepper_30.bmp]

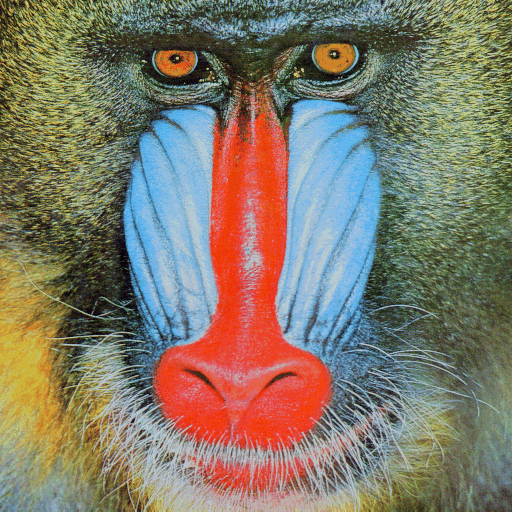

Supplement: Supplemental Information 5 — Copyright 2019 Mohammad Shamim Imtiaz. [file peerj-cs-08-1115-s005.zip › Stego_image_Lena_Baboon_Pepper/PVD_stego_Baboon 44000.bmp]

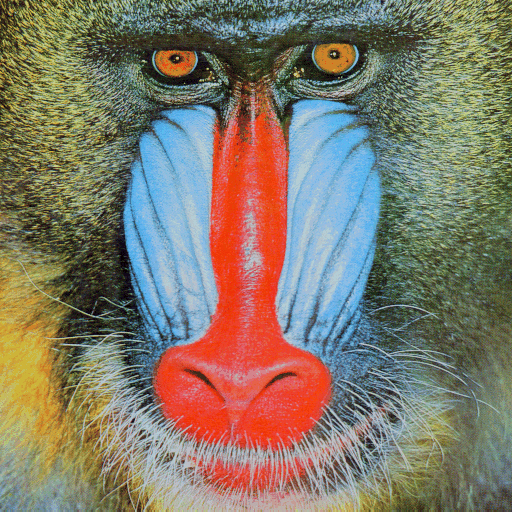

Supplement: Supplemental Information 5 — Copyright 2019 Mohammad Shamim Imtiaz. [file peerj-cs-08-1115-s005.zip › Stego_image_Lena_Baboon_Pepper/PVD_stego_Baboon 36000.bmp]

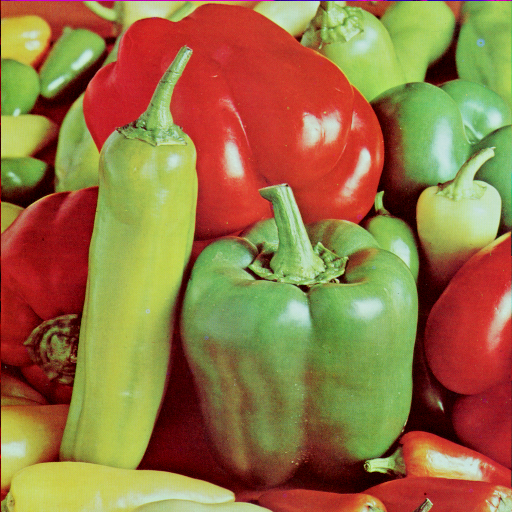

Supplement: Supplemental Information 5 — Copyright 2019 Mohammad Shamim Imtiaz. [file peerj-cs-08-1115-s005.zip › Stego_image_Lena_Baboon_Pepper/PVD_stego_Pepper 60000.bmp]

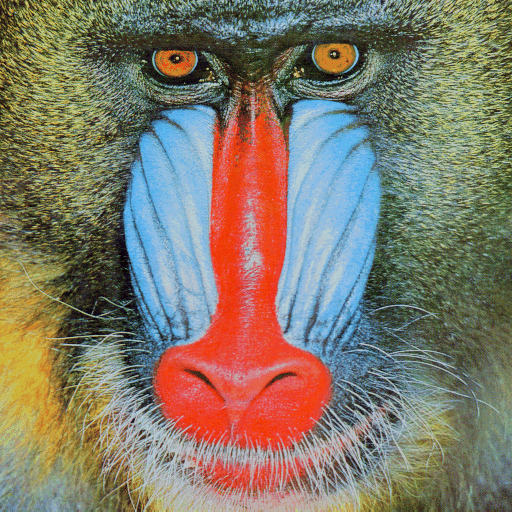

Supplement: Supplemental Information 5 — Copyright 2019 Mohammad Shamim Imtiaz. [file peerj-cs-08-1115-s005.zip › Stego_image_Lena_Baboon_Pepper/PVD_stego_Baboon 56000.bmp]

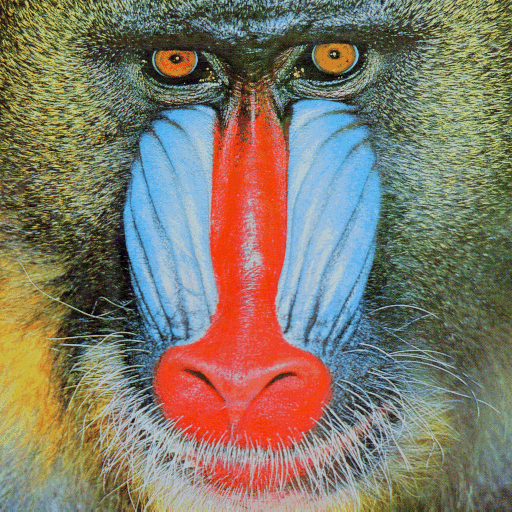

Supplement: Supplemental Information 5 — Copyright 2019 Mohammad Shamim Imtiaz. [file peerj-cs-08-1115-s005.zip › Stego_image_Lena_Baboon_Pepper/PVD_stego_Baboon 28000.bmp]

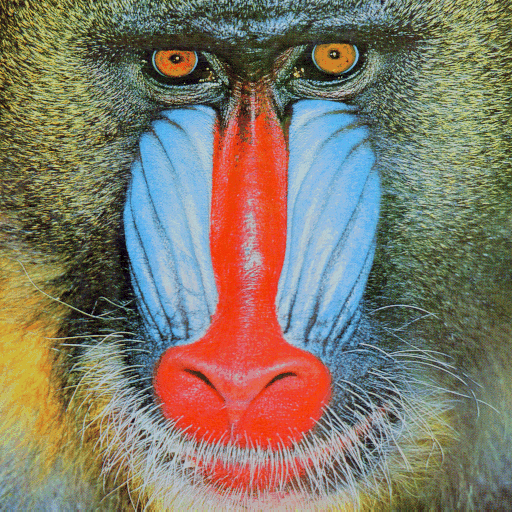

Supplement: Supplemental Information 5 — Copyright 2019 Mohammad Shamim Imtiaz. [file peerj-cs-08-1115-s005.zip › Stego_image_Lena_Baboon_Pepper/PVD_stego_Baboon 20000.bmp]

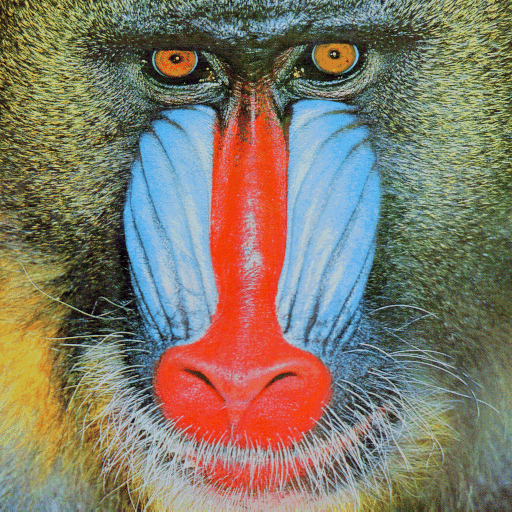

Supplement: Supplemental Information 5 — Copyright 2019 Mohammad Shamim Imtiaz. [file peerj-cs-08-1115-s005.zip › Stego_image_Lena_Baboon_Pepper/PVD_stego_image_Baboon_10.bmp]

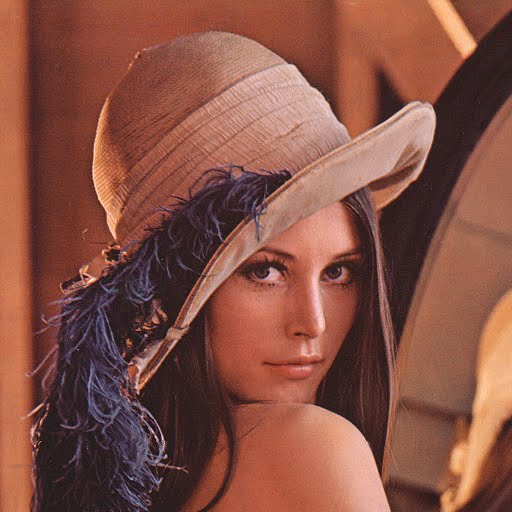

Supplement: Supplemental Information 5 — Copyright 2019 Mohammad Shamim Imtiaz. [file peerj-cs-08-1115-s005.zip › Stego_image_Lena_Baboon_Pepper/PVD_stego_image_Pepper10.bmp]
