# Supplementary material for: New deep data hiding and extraction algorithm using multi-channel with multi-level to improve data security and payload capacity
Source: PeerJ Comput Sci. 2022 Oct 19;8:e1115. doi: 10.7717/peerj-cs.1115 (PMC9680874; doi:10.7717/peerj-cs.1115)
Supplement: Supplemental Information 11 [file peerj-cs-08-1115-s011.docx]

The implementation of hiding algorithm (MCDHA) using Matlab Programing language:

Encoding Function:

This function takes the Secret message, Cover image and the encryption key as input and returns the stego image.

| function [J,SN]=PVD_RGB_Code(I,text_file,enc_key)  e1=0;  e2=0;  e3=0;    msgtype = ischar(text_file); % If message is text this will be true;  if msgtype == 1 % Message = TEXT  msg_temp = double(text_file); % Converts from ASCII to Integer Values.  msg_dim = num2str(length(msg_temp));  msg_length = length(msg_dim);  z = 0;  if msg_length < 7  padtext = 7 - msg_length;  for z = 1:padtext  msg_dim = horzcat('0',msg_dim);  end  msg_head = horzcat('t',msg_dim);  % Applying Header To Beginning of Message to be Encoded.  msg_temp_head = horzcat(msg_head,msg_temp);  end  else  % Message = IMAGE  msg_temp = im2uint8(text_file); % Convert to Integer Value Representation.  % Determine Message Image's Size for Encoding in Header  [hideM1,hideN1] = size(msg_temp);  hideM = num2str(hideM1);  hideN = num2str(hideN1);  dimM = length(hideM);  dimN = length(hideN);  padM = 0; padN = 0;  z = 0;    if dimM < 4  padM = 4 - dimM;  for z = 1:padM  % Zero Padding Dimension if less than 4 Sig Figs.  hideM = horzcat('0',hideM);  end  end  z = 0;    if dimN < 4  padN = 4 - dimN;  for z = 1:padN  % Zero Padding Dimension if less than 4 Sig Figs.  hideN = horzcat('0',hideN);  end  end  msg_head = horzcat(hideM,hideN);  msg_temp_head = msg_head;    y = 0; k = hideM1;  for y = 1:k  % Applying Header To Beginning of Message to be Encoded.  msg_temp_head = horzcat(msg_temp_head,msg_temp(y,:));  end    end    %text_read_ascii =uint8(msg_temp_head);  text_read_ascii = bitxor(uint8(msg_temp_head),uint8(enc_key));    binaryString = transpose(de2bi(text_read_ascii,8));  size_binaryString = size(binaryString);  final_message = reshape(binaryString,1,size_binaryString(1)*size_binaryString(2));  final_message(length(final_message)+1:length(final_message)) =[0];    i1 = 1;  S=double(I);  SN=0;  TNOB=0;B=S;  SN=0;  TNOB=0;  for i=1:size(S,1)-2 %Sobel operator for edge detection  for j=1:size(S,2)-2  Gx=((2*S(i+2,j+1,1)+S(i+2,j,1)+S(i+2,j+2,1))-(2*S(i,j+1,1)+S(i,j,1)+S(i,j+2,1)));  Gy=((2*S(i+1,j+2,1)+S(i,j+2,1)+S(i+2,j+2,1))-(2*S(i+1,j,1)+S(i,j,1)+S(i+2,j,1)));  B(i,j,1)=sqrt(Gx.^2+Gy.^2);  end  end  for i=1:size(S,1)-2 %Sobel operator for edge detection  for j=1:size(S,2)-2  Gx=((2*S(i+2,j+1,2)+S(i+2,j,2)+S(i+2,j+2,2))-(2*S(i,j+1,2)+S(i,j,2)+S(i,j+2,2)));  Gy=((2*S(i+1,j+2,2)+S(i,j+2,2)+S(i+2,j+2,2))-(2*S(i+1,j,2)+S(i,j,2)+S(i+2,j,2)));  B(i,j,2)=sqrt(Gx.^2+Gy.^2);  end  end  for i=1:size(S,1)-2 %Sobel operator for edge detection  for j=1:size(S,2)-2  Gx=((2*S(i+2,j+1,3)+S(i+2,j,3)+S(i+2,j+2,3))-(2*S(i,j+1,3)+S(i,j,3)+S(i,j+2,3)));  Gy=((2*S(i+1,j+2,3)+S(i,j+2,3)+S(i+2,j+2,3))-(2*S(i+1,j,3)+S(i,j,3)+S(i+2,j,3)));  B(i,j,2)=sqrt(Gx.^2+Gy.^2);  end  end  B=uint8(B);  while(i1 <= 254)  for j1 = 1:1:254  %% compute number of bit per pixel in red channel  inner_loop=0;  v=0;  for x1=i1:i1+2  for x2=j1:j1+2  inner_loop=inner_loop+1;  %if(mod(inner_loop,2)==0)  %if(inner_loop~=5)  v=v+S(x1,x2,1);    % end  end  end  Mi=v/9;  sum=0;  inner_loop=0;  for x1=i1:i1+2  for x2=j1:j1+2  inner_loop=inner_loop+1;  %if(mod(inner_loop,2)==0)  %if(inner_loop~=5)  sum=sum+((S(x1,x2,1)-Mi).^2);    %end  if(inner_loop ==5)  u1=x1;  u2=x2;    end  end  end  segma=sqrt((sum)/9);  if(B(u1,u2,1)>250)  NBPP=3;  e3=e3+1;  elseif(segma <= 6)  NBPP=1;  e1=e1+1;  elseif(segma > 6 && segma<=18)  NBPP=2;  e2=e2+1;  else  NBPP=3;  e3=e3+1;  end    TNOB=TNOB+NBPP;  disp(NBPP);  pixel_count = 0;  for i = i1:i1+2  for j = j1:j1+2  pixel_count = pixel_count+1;  if(pixel_count==5)  A1=i;  A2=j;  old=S(i,j,1);    binary_form=de2bi(old,8);  if(length(final_message)==0)  break  end  if(length(final_message)>=1 && length(final_message)<NBPP)  final_message(length(final_message)+1:NBPP)=[0];  end    binary_form(NBPP:-1:1)=final_message(1:NBPP);  final_message(1:NBPP)=[];  new=bi2de(binary_form);  S(i,j,1)=new;  SN(i,j,1)=NBPP;  end  end  if(length(final_message)==0)  break  end  end    if(length(final_message)==0)  break  end    %% compute number of bit per pixel in Green channel  inner_loop=0;  v=0;  for x1=i1:i1+2  for x2=j1:j1+2  inner_loop=inner_loop+1;  %if(mod(inner_loop,2)==0)  %if(inner_loop~=5)  v=v+S(x1,x2,2);    %end  end  end  Mi=v/9;  sum=0;  inner_loop=0;  for x1=i1:i1+2  for x2=j1:j1+2  inner_loop=inner_loop+1;  %if(mod(inner_loop,2)==0)  %if(inner_loop~=5)  sum=sum+((S(x1,x2,2)-Mi).^2);  if(inner_loop ==5)  u1=x1;  u2=x2;  end  end  end    segma=sqrt((sum)/9);  if(B(u1,u2,1)>250)  NBPP=3;  e3=e3+1;  elseif(segma <= 6)  NBPP=1;  e1=e1+1;  elseif(segma > 6 && segma<=18)  NBPP=2;  e2=e2+1;  else  NBPP=3;  e3=e3+1;  end    TNOB=TNOB+NBPP;  disp(NBPP);  pixel_count = 0;  for i = i1:i1+2  for j = j1:j1+2  pixel_count = pixel_count+1;  if(pixel_count==5)  A1=i;  A2=j;  old=S(i,j,2);  binary_form=de2bi(old,8);  if(length(final_message)==0)  break  end  if(length(final_message)>=1 && length(final_message)<NBPP)  final_message(length(final_message)+1:NBPP)=[0];  end    binary_form(NBPP:-1:1)=final_message(1:NBPP);  final_message(1:NBPP)=[];  new=bi2de(binary_form);  S(i,j,2)=new;  SN(i,j,2)=NBPP;  end  end  if(length(final_message)==0)  break  end  end    if(length(final_message)==0)  break  end  %% compute number of bit per pixel in Blue channel  inner_loop=0;  v=0;  for x1=i1:i1+2  for x2=j1:j1+2  inner_loop=inner_loop+1;  %if(mod(inner_loop,2)==0)  %if(inner_loop~=5)  v=v+S(x1,x2,3);    % end  end  end  Mi=v/9;  sum=0;  inner_loop=0;  for x1=i1:i1+2  for x2=j1:j1+2  inner_loop=inner_loop+1;  %if(mod(inner_loop,2)==0)  %if(inner_loop~=5)  sum=sum+((S(x1,x2,3)-Mi).^2);  if(inner_loop ==5)  u1=x1;  u2=x2;    end  end  end    segma=sqrt((sum)/9);  if(B(u1,u2,1)>250)  NBPP=3;  e3=e3+1;  elseif(segma <= 6)  NBPP=1;  e1=e1+1;  elseif(segma > 6 && segma<=18)  NBPP=2;  e2=e2+1;  else  NBPP=3;  e3=e3+1;  end  TNOB=TNOB+NBPP;  disp(NBPP);  pixel_count = 0;  for i = i1:i1+2  for j = j1:j1+2  pixel_count = pixel_count+1;  if(pixel_count==5)  A1=i;  A2=j;  old=S(i,j,3);    binary_form=de2bi(old,8);  if(length(final_message)==0)  break  end  if(length(final_message)>=1 && length(final_message)<NBPP)  final_message(length(final_message)+1:NBPP)=[0];  end    binary_form(NBPP:-1:1)=final_message(1:NBPP);  final_message(1:NBPP)=[];  new=bi2de(binary_form);  S(i,j,3)=new;  SN(i,j,3)=NBPP;  end  end  if(length(final_message)==0)  break  end  end    if(length(final_message)==0)  break  end  end    if(length(final_message)==0)  break  end    i1=i1+1;  end  if(length(final_message)~=0)  disp('Error Size');  end  J=uint8(S);  disp(e1);  disp(e2);  disp(e3);    imwrite(J,'PVD_stego_image.bmp');  end |
| --- |
|  |
| function [ci]=decomp(X,dict,Image)  decodedVal = huffmandeco(X,dict);  decodedVal = uint8(decodedVal);  [rows, columns, numberOfColorChannels] = size(Image);  ci = reshape(decodedVal,[rows, columns, numberOfColorChannels]) ;  imwrite(ci,'decoded.png');  end |
|  |
| function [encodedVal,dict]=comp(Image)  % calculate the frequency of each pixel  [frequency,pixelValue] = imhist(Image());    % sum all the frequencies  tf = sum(frequency) ;    % calculate the frequency of each pixel  probability = frequency ./ tf ;    % create a dictionary  dict = huffmandict(pixelValue,probability);    % get the image pixels in 1D array  imageOneD = Image(:) ;    % encoding  testVal = imageOneD ;  encodedVal = huffmanenco(testVal,dict);  end |
|  |
| function ca=splitRGB(img)  rgbImage=img;    %set(gcf, 'units','normalized','outerposition',[0 0 1 1]);  %drawnow;  [rows columns numberOfColorBands] = size(rgbImage);  % The first way to divide an image up into blocks is by using mat2cell().  blockSizeR = rows/2; % Rows in block.  blockSizeC = columns; % Columns in block.  wholeBlockRows = floor(rows / blockSizeR);  blockVectorR = [blockSizeR * ones(1, wholeBlockRows)];  wholeBlockCols = floor(columns / blockSizeC);  blockVectorC = [blockSizeC * ones(1, wholeBlockCols)];  ca = mat2cell(rgbImage, blockVectorR, blockVectorC, numberOfColorBands);  end |
|  |
| function ca=splitGray(img)  rgbImage=img;  imshow(rgbImage);  %set(gcf, 'units','normalized','outerposition',[0 0 1 1]);  %drawnow;  [rows columns] = size(rgbImage);  % The first way to divide an image up into blocks is by using mat2cell().  blockSizeR = rows/2; % Rows in block.  blockSizeC = columns; % Columns in block.  wholeBlockRows = floor(rows / blockSizeR);  blockVectorR = [blockSizeR * ones(1, wholeBlockRows)];  wholeBlockCols = floor(columns / blockSizeC);  blockVectorC = [blockSizeC * ones(1, wholeBlockCols)];  ca = mat2cell(rgbImage, blockVectorR, blockVectorC);  end |
|  |
| function k=joinRGB(sub1,sub2)  [r1,c1,ch]=size(sub1);  [r2,c2,ch]=size(sub2);  k=uint8(ones(r1+r2,c1));  k(1:r1,:,1)=sub1(:,:,1);  k(1:r1,:,2)=sub1(:,:,2);  k(1:r1,:,3)=sub1(:,:,3);  k(r1+1:end,:,1)=sub2(:,:,1);  k(r1+1:end,:,2)=sub2(:,:,2);  k(r1+1:end,:,3)=sub2(:,:,3);  imshow(k);  end |

The implementation of extraction algorithm (MCDEA) using Matlab Programing language:

Decoding Function:

| function PVD_RGB_Decode(J,SN,enc_key)  [N, M, ch]=size(SN);  M=M-1;  S=double(J);  final_msg=[];  msg='';  ccc=0;  i1=1;  while(i1<N)  for j1=1:1:M  pixel_count = 0;  for i = i1:i1+2  for j = j1:j1+2  pixel_count = pixel_count+1;  if(pixel_count==5)  A1=i;  A2=j;  m1=de2bi(S(i,j,1),8);  NBPP=SN(i,j,1);  for loop=NBPP:-1:1  msg=strcat(msg,num2str(m1(loop)));  end  end  end  end  %***** Green Channel  pixel_count = 0;  for i = i1:i1+2  for j = j1:j1+2  pixel_count = pixel_count+1;  if(pixel_count==5)  A1=i;  A2=j;  m1=de2bi(S(i,j,2),8);  NBPP=SN(i,j,2);  for loop=NBPP:-1:1  msg=strcat(msg,num2str(m1(loop)));  end  end  end  end  %******* Blue Channel  pixel_count = 0;  for i = i1:i1+2  for j = j1:j1+2  pixel_count = pixel_count+1;  if(pixel_count==5)  A1=i;  A2=j;  m1=de2bi(S(i,j,3),8);  NBPP=SN(i,j,3);  for loop=NBPP:-1:1  msg=strcat(msg,num2str(m1(loop)));  end  end  end  end  end    i1=i1+1;  end    len_msg=floor(length(msg)/8);  for outer_loop=1:len_msg  ccc=ccc+1;  final_msg(outer_loop)=bin2dec(fliplr(msg(ccc:ccc+7)));  final_msg(outer_loop)= bitxor(uint8(final_msg(outer_loop)),uint8(enc_key));  ccc=ccc+7;  end    %% ************************************************    if final_msg(1) == 116  %% CASE 1: Text Set  dim1 = char(final_msg(2:8));  m = str2double(dim1');  n = 1;    msg_set = final_msg(9:end);  final_msg= char(msg_set');  outlet = char(final_msg);  fid = fopen('PVD_Extract.txt','w');  fun = fprintf(fid,'%c',final_msg);  fclose(fid);  else  %% CASE 2: Image Set  % Determine Dimensions from Header Values  tempm = char(final_msg(1:4));  tempn = char(final_msg(5:8));  m = str2double(tempm');  n = str2double(tempn');  % CASE 2: Image Set  % Determine Dimensions from Header Values      msg_set = final_msg(9:end);    count = 1;  msg_out = uint8(zeros(m,n));  for y = 1:m  for x = 1:n  msg_out(y,x) = msg_set(count);  count = count + 1;  end  end  msg_out = im2uint8(msg_out);  end  imshow( msg_out);  end |
| --- |

Testing Function:

1. Mean Square Error and Peak signal to noise ratio

| function MSE_PSN(I,J3)  %display MSE  origImg = double(I);  distImg = double(J3);  [D F] = size(origImg);  error = origImg - distImg;  MSE = sum(sum(error .* error)) / (D * F);  mse = sprintf('MSE is: %0.6f\n',MSE);  disp(mse)  %%  %display PSNR  for j=1 :length(MSE)    if(MSE > 0)    PSNR = 10*log(255*255/MSE) / log(10);    else    PSNR = 99;  end  psnr = sprintf('PSNR is: %0.6f\n',PSNR);  disp(psnr)  end  %% |
| --- |

1. Normalized Cross Correlation

| function NCC(CI,SI)  CI=CI-mean(CI(:));  SI=SI-mean(SI(:));  denom=sqrt(sum(sum(CI.^2)).*sum(sum(SI.^2)));  m=sum(sum((CI.*SI)))./denom;  disp(m) |
| --- |

1. Structural similarity index measure:

| %% Structural similarity (SSIM) index for measuring image quality  [ssimval,ssimmap] = ssim(J3,I);  disp(ssimval);  %% |
| --- |

1. Dissimilarity between adjacent pixel

| function pixel_Difference(I,J)  figure;  II=double(I);    VDV=diff(II,1,1);  CI=rgb2gray(VDV);  CII=imhist(CI);  y1=histogram(VDV,'BinLimits',[-40,40],'FaceColor','r');  y1.BinWidth = 1;  I2=double(J);  VDV=diff(I2,1,1);  CI=rgb2gray(VDV);  CII=imhist(uint8(CI));  hold on  y2=histogram(VDV,'BinLimits',[-40,40],'FaceColor','b') ;  y2.BinWidth = 1;  y2.EdgeColor = 'b';    title('Baboon 512x512');  xlabel('pixel difference');  ylabel('Frequency');  legend({'Cover Image','Stego Image'},'Location','southoutside'); |
| --- |

1. Euclidean norm test

| Euclidean_norm_test(J3,I)  R1 = I(:,:,1); R2 = J3(:,:,1);  G1 = I(:,:,2); G2 = J3(:,:,2);  B1 =I(:,:,3); B2 = J3(:,:,3);  s = (R1-R2).^2+(G1-G2).^2+(B1-B2).^2;  s = s(:);  d= sqrt(sum(s));  disp(d); |
| --- |
